# Our re-do extirpation in a case with recurrent giant left atrial myxoma forming mitral stenosis

**DOI:** 10.1186/1749-8090-8-S1-P170

**Published:** 2013-09-11

**Authors:** U Yetkin, E Celik, I Yurekli, A Gurbuz

**Affiliations:** 1 Department of Cardiovascular Surgery, Izmir Katip Celebi University Ataturk Training and Research Hospital, Izmir, Turkey

## Background

Myxoma is the most common primary benign cardiac tumor of the adulthood. It usually originates from the fossa ovalis area of the interatrial septum. The sensitivity of 2-D TTE is optimal as a diagnostic tool. The treatment modality of the myxomas is surgery. The recurrence of the myxoma is rare.

## Methods

Our case was a 41-year-old female. She had undergone a successful extirpation of a left atrial myxoma with a partial resection and primary repair of the interatrial septum 2 years ago. She was suffering from dyspnea and fatigue for 1 month. Her TTE revealed a mass lesion of 49x22 mm within the left atrial cavity originating from the interatrial septum, consistent with a recurrent myxoma.

## Results

She was emergently taken into operating room to avoid major and even fatal complications as systemic embolization and sudden cardiac death. After right atriotomy an incision was made to the fossa ovalis vertically and superiorly. Interatrial septum was opened. The area of the interatrial septum where the recurrent left atrial myxoma originated from was resected with the total extirpation of the myxoma. No complication was seen in intensive care unit and ward follow-up. We carefully investigated our case in terms of Carney complex including familial myxoma and endocrine pathologies. Late period TTE showed no recurrence. Outpatient follow-up continues event-free.

## Conclusions

Atrial myxoma cases should be operated as soon as possible due to high embolization risks and its potential for developing fatal complications as sudden cardiac death. In order to avoid its recurrence, the interatrial septum where the myxomatous tissue originated from should be widely excised and the septum should be closed accordingly. The transseptal approach -as we did- allows an optimal surgical exposure and easy extirpation of the tumor. We think that annual TTE examination should be performed within first 10 years to detect early or late period recurrences.

